# Recent Advances in Marine-Derived Compounds as Potent Antibacterial and Antifungal Agents: A Comprehensive Review

**DOI:** 10.3390/md22080348

**Published:** 2024-07-29

**Authors:** Devaraj Bharathi, Jintae Lee

**Affiliations:** School of Chemical Engineering, Yeungnam University, 280 Daehak-Ro, Gyeongsan 38541, Republic of Korea

**Keywords:** antibacterial activity, antifungal activity, bioactive compounds, marine-derived drugs, therapeutic applications

## Abstract

The increase in antimicrobial resistance (AMR) in microorganisms is a significant global health concern. Various factors contribute to AMR, including alterations in cell membrane permeability, increased efflux pump activity, enzymatic modification or inactivation of antibiotics, target site changes, alternative metabolic pathways, and biofilm formation. Marine environments, with their extensive biodiversity, provide a valuable source of natural products with a wide range of biological activities. Marine-derived antimicrobial compounds show significant potential against drug-resistant bacteria and fungi. This review discusses the current knowledge on marine natural products such as microorganisms, sponges, tunicates and mollusks with antibacterial and antifungal properties effective against drug-resistant microorganisms and their ecological roles. These natural products are classified based on their chemical structures, such as alkaloids, amino acids, peptides, polyketides, naphthoquinones, terpenoids, and polysaccharides. Although still in preclinical studies, these agents demonstrate promising in vivo efficacy, suggesting that marine sources could be pivotal in developing new drugs to combat AMR, thereby fulfilling an essential medical need. This review highlights the ongoing importance of marine biodiversity exploration for discovering potential antimicrobial agents.

## 1. Introduction

**Figure 1:**
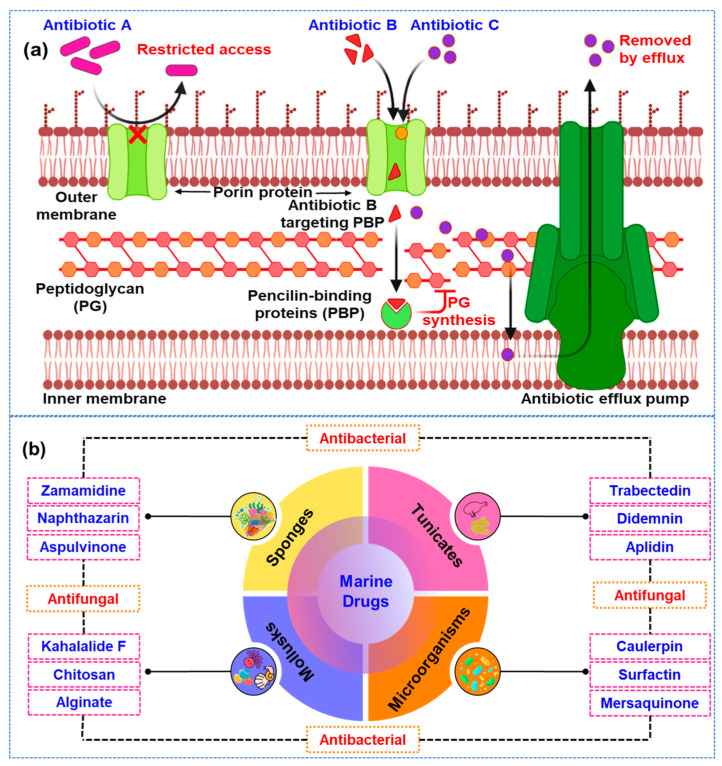
(**a**) The antibiotic resistance mechanisms of currently available antibiotics against pathogenic microorganisms. The image, created with BioRender.com (accessed on 25 May 2024), illustrates various mechanisms by which pathogens develop resistance, including alteration of antibiotic targets, enzymatic degradation of antibiotics, and efflux pump activation. Each mechanism is depicted with representative examples of affected antibiotics and the corresponding microbial adaptations, (**b**) Different sources of marine drugs with their potential compounds for antibacterial and antifungal properties. These marine-derived compounds play a crucial role in the development of new therapeutic agents to combat microbial infections.

Antimicrobial resistance (AMR) is an escalating global health issue, significantly undermining the efficacy of current antibiotics and increasing the risk of untreatable infections [1,2,3]. This problem stems from the widespread overuse and misuse of antibiotics in both medical and agricultural settings, leading to the emergence of multidrug-resistant (MDR) pathogens [4,5]. The World Health Organization (WHO) projects that, without intervention, AMR could cause up to 10 million deaths annually by 2050 [6]. Bacteria can develop resistance through several mechanisms, including changes in cell membrane permeability, activation of efflux pumps, enzymatic degradation of antibiotics, modifications to target sites, alternative metabolic pathways, and biofilm formation [7,8,9]. The antibiotic resistance mechanism of currently available antibiotics against microbial pathogens is shown in Figure 1a. These strategies collectively reduce the effectiveness of traditional antimicrobial treatments.

The vast and largely untapped biodiversity of marine environments offers a promising avenue for discovering new antimicrobial agents [10]. Marine organisms, especially microorganisms, have evolved unique biochemical pathways to survive the extreme conditions of their habitats. These environments include high pressure, low light, varying temperatures, and high salinity, which have driven marine life to develop novel adaptations [11,12]. As a result, these organisms produce a wide array of bioactive compounds not typically found in terrestrial environments. Marine microorganisms, in particular, have become a focal point of research due to their ability to synthesize secondary metabolites with remarkable biological activities [13,14]. These secondary metabolites are not directly involved in the normal growth, development, or reproduction of the organism. Instead, they often assist ecological functions, such as defense mechanisms against predators, competition with other microorganisms, or communication within their communities [15]. The unique properties of these compounds have garnered significant interest for their potential therapeutic applications. Among the various bioactive compounds produced by marine microorganisms, several classes stand out for their antimicrobial properties [16]. For example, peptides are short chains of amino acids that can disrupt microbial cell membranes, leading to cell death [17,18]. Polyketides are another class of compounds known for their structural diversity and potent biological activities [19]. Alkaloids, which contain nitrogen atoms, have been found to possess a range of pharmacological effects, including antimicrobial activity [20]. Terpenoids, derived from isoprene units, and polysaccharides are long carbohydrate molecules, which also contribute to the arsenal of bioactive compounds with potential antimicrobial applications (Figure 1b) [21,22].

Recent research has increasingly turned to marine microbial compounds for potential antibiotics. This shift is driven by the unique, high-stress environments of marine habitats, which promote the production of metabolites distinct from those in terrestrial organisms, and advancements in bioprospecting technologies that enable the collection of samples from previously inaccessible areas like deep-sea vents and polar regions [23,24,25]. For example, secondary metabolites from marine-derived *Streptomyces* sp. have shown significant activity against drug-resistant strains of *Enterococcus faecium*, *Staphylococcus aureus*, and *Mycobacterium tuberculosis* [26,27]. In the fight against AMR, exploring marine natural products is particularly critical due to the urgent need for new antimicrobial agents with unique mechanisms of action [28]. Compounds such as nocardiopsistins, stremycins, and chlororesistoflavins from marine sources have demonstrated effectiveness against methicillin-resistant *S. aureus* (MRSA), vancomycin-resistant Enterococci (VRE), and multidrug-resistant *M. tuberculosis* (MDR-TB) [26,27,29]. These marine-derived compounds not only offer new mechanisms of action but also possess structural features that are absent in conventional antibiotics, making them less likely to encounter cross-resistance [30,31]. The exploration and characterization of such compounds are essential for expanding our target of antimicrobial agents and providing unique solutions to combat AMR.

Marine compounds contain unusual ring systems, halogenated compounds, highly branched molecules, and sulfated polysaccharides, which offer different modes of action compared to traditional antibiotics and also it can reduce the likelihood of cross-resistance [30]. Halogen atoms are able to establish non-covalent interactions with biomolecules, ensuring binding, specificity and enhanced drug efficacy against AMR pathogens [1]. Some marine-derived compounds can also enhance the efficacy of existing antibiotics by inhibiting bacterial efflux pumps, a common resistance mechanism. This synergistic effect not only restores the potency of conventional antibiotics but also broadens their spectrum of activity. For instance, an antibiotic that was previously ineffective against a particular strain of bacteria due to resistance mechanisms might regain its effectiveness when used in combination with a marine-derived efflux pump inhibitor [32,33].

While previous reviews address the isolation of marine-derived compounds for antibacterial and antifungal activities [12,13,14,15,16,21,24,25,26], this review stands out by focusing on recent research articles highlighting the extraction or isolation of marine-derived compounds with potential antimicrobial activity against multidrug-resistant (MDR) pathogens. Unlike previous reviews that focus on a single type of compound from specific marine sources, this review provides a detailed summary of compounds derived from various marine sources. It spans recent advancements and research articles from approximately 2010 to 2024, highlighting significant progress in marine-derived compounds as potent antibacterial and antifungal agents. This paper emphasizes the vast potential of marine biodiversity, showcasing marine-derived compounds with unique structures and mechanisms of action against resistant pathogens. It categorizes these compounds based on their chemical structures, including alkaloids, amino acids, peptides, polyketides, naphthoquinones, terpenoids, and polysaccharides, each with distinct mechanisms of action. Additionally, this review details how the unique chemical structures of marine-derived compounds result in different modes of action compared to traditional antibiotics. Furthermore, this review explores the connection between the antimicrobial properties of these compounds and their ecological roles, providing insights into their evolutionary significance and potential applications. Also, this review highlights significant progress and discoveries in marine-derived compounds as potent antibacterial and antifungal agents.

## 2. Marine-Derived Alkaloids

**Figure 2:**
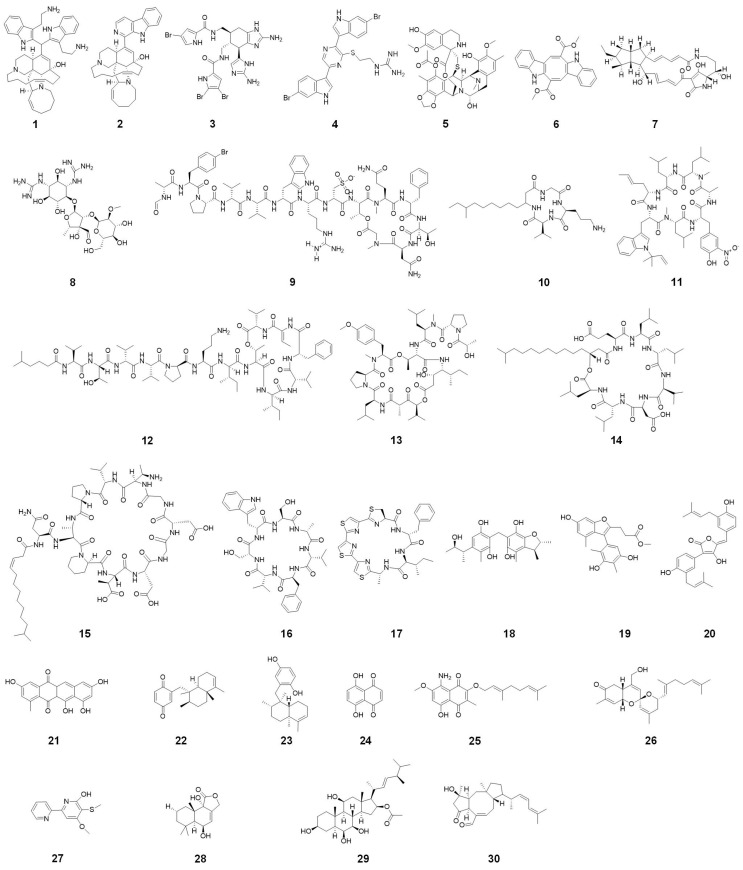
Chemical structures of zamamidine D (**1**), manzamine A (**2**), bromoageliferin (**3**), dragmacidin G (**4**), trabectedin (**5**), caulerpin (**6**), alteramide A (**7**), streptomycin (**8**), halicylindramide A (**9**), rhodopeptins C1 (**10**), ilamycin B1 (**11**), kahalalide F (**12**), didemnin B (**13**), surfactin (**14**), friulimicin B (**15**), asperversiamide A (**16**), marthiapeptide A (**17**), dicitrinone E (**18**), talarominine A (**19**), aspulvinones H (**20**), mersaquinone (**21**), avarone (**22**), avarol (**23**), 5,8-Dihydroxy-1,4-naphthoquinone (**24**), flaviogeranin B (**25**), phorbaketal A (**26**), collismycin C (**27**), strobilactone A (**28**), penicisteroid A (**29**), and ophiobolin K (**30**).

Marine organisms, such as sponges, tunicates, mollusks, algae, and microorganisms like bacteria and fungi, generate a diverse range of alkaloids [34,35,36,37,38]. These compounds have evolved as part of the organisms’ defense mechanisms, resulting in the unique structures and potent biological activities. Sponges are a prolific source of bioactive compounds, most notably alkaloids, which have garnered significant attention for their potent antibacterial and antifungal activities [39,40]. These sessile invertebrates inhabit diverse marine environments, from shallow reefs to the deep sea, where they have evolved complex chemical defense mechanisms to survive against a myriad of microbial threats. The unique structural diversity of sponge-derived alkaloids underpins their broad-spectrum bioactivity, making them promising candidates for developing new antimicrobial agents [41]. The chemical structure of various marine-derived alkaloids is shown in Figure 2.

Takaaki Kubota et al. [42] demonstrated the isolation and analysis of zamamidine D (**1**). Compound **1** was obtained at a yield of 1.7 mg from 0.68 kg of wet sponge material, which corresponds to 0.00025% of the wet weight. The antimicrobial assays showed that zamamidine D is highly potent, with an MIC of 0.032 mg/mL for *E. coli*, and 0.008 mg/mL for *S. aureus*, *B. subtilis*, and *M. luteus*, and IC_50_ values of 0.016 mg/mL for *C. albicans* and *A. niger*, and 0.002 mg/mL for *C. neoformans*. These results underscore the compound’s significant antimicrobial potential. Other notable sponge-derived alkaloids include manzamine A (**2**) [43], which has shown promising antibacterial activity against *M. tuberculosis*. Research by Rateb et al. [44] underscores the potential of manzamine A as a lead compound for developing new anti-TB drugs. Alkaloids isolated from marine sponges exhibit a wide range of chemical structures, including pyrrole, quinoline, isoquinoline, and indole derivatives, each contributing to their potent biological activities [45,46]. These compounds are often synthesized as secondary metabolites, serving as chemical defenses to protect the sponges from pathogenic microorganisms and predators. This evolutionary pressure has resulted in the production of alkaloids with highly specialized and effective antimicrobial properties [47,48].

Recently, Pech-Puch et al. [49] isolated eight alkaloids from the sponge *Agelas dilatata*, exhibiting potent antibacterial activity. Bromoageliferin (**3**) notably exhibited strong antibacterial effects against *P. aeruginosa*. Quantitative analyses showed that the MICs for bromoageliferin against different strains of *P. aeruginosa* ranged from 0.008 to 0.032 mg/mL, highlighting its potency. These results indicate that bromoageliferin holds promise as a lead compound for developing new antibacterial treatments targeting multidrug-resistant pathogens.

The pharmaceutical potential of sponge-derived alkaloids extends beyond their antimicrobial properties. The unique chemical structures of these compounds provide a scaffold for the progress of novel drugs with better efficacy and reduced resistance. For instance, researchers have been exploring the synthetic modification of sponge alkaloids to enhance their stability, bioavailability, and selectivity for microbial targets, paving the way for new therapeutic applications. A study by Hong et al. [50] discusses the potential for synthetic chemistry to unlock new derivatives of sponge alkaloids with enhanced pharmacological properties.

Moreover, the ecological role of alkaloids in marine sponges underscores their importance in maintaining marine biodiversity and health. By producing bioactive compounds, sponges contribute to the regulation of microbial populations in their habitats, preventing the overgrowth of harmful pathogens and promoting a balanced ecosystem. This ecological function highlights the potential benefits of preserving marine biodiversity, as the loss of sponge species could mean the loss of valuable bioactive compounds with significant medical applications [51]. Leal et al. [52] emphasize the ecological significance of sponge-derived alkaloids and the need for conservation efforts to protect these valuable marine resources.

Recent advancements in marine biotechnology have facilitated the sustainable extraction and synthesis of sponge alkaloids, reducing the environmental impact of bioprospecting. Advances in aquaculture and microbial fermentation techniques are enabling the large-scale production of these compounds, making them more accessible for pharmaceutical research and development. A review by Li et al. [53] discusses the potential of microbial symbionts in sponges as an alternative source of bioactive alkaloids, highlighting innovative approaches to sustainable drug discovery. Compound **4** shown in Figure 2 is dragmacidin G. Dragmacidin G isolated from a deep-water sponge of the genus *Spongosorties* exhibited broad-spectrum antibacterial activity against MRSA and *M. tuberculosis* [54]. 

Marine tunicates, commonly known as sea squirts, are a notable source of bioactive alkaloids with significant antibacterial and antifungal properties [55,56]. Prominent tunicate-derived alkaloids include trabectedin (**5**), which has been noted for its antifungal activity, highlighting its potential for new antifungal therapeutics [57]. Additionally, Blunt et al. [40] discussed the ecological importance of these alkaloids and their role in the chemical defense of tunicates, reinforcing the value of marine biodiversity in discovering new bioactive compounds. Moreover, researchers explored the biosynthetic pathways of tunicate alkaloids, providing insights into their complex structures and potential applications in synthetic biology. Recently, several researchers demonstrated the use of microbial fermentation techniques to sustainably produce marine-derived alkaloids, which reduces the environmental impact typically associated with traditional bioprospecting. Their study highlights the potential of using engineered microorganisms to replicate the biosynthesis pathways of these marine compounds, thus enabling large-scale production without the need to harvest marine organisms directly [58,59].

Additionally, Xiong et al. [60] reviewed the potential of genome mining to identify new alkaloid-producing genes, highlighting innovative strategies for drug discovery. These advancements not only improve access to these compounds for pharmaceutical research but also contribute to marine ecosystem conservation [61]. Continued exploration of marine alkaloids holds great promise for developing novel antibacterial and antifungal agents, addressing the urgent need for new treatments in combating infectious diseases.

Marine microorganisms, including algae, bacteria, and fungi, are prolific producers of bioactive alkaloids with significant antibacterial and antifungal properties. Algae-derived alkaloids, particularly from green algae like *Caulerpa* sp., have demonstrated remarkable antibacterial activity against several pathogens, including multi-drug resistant strains. For instance, caulerpin (**6**), an indole alkaloid derived from *Caulerpa*, has demonstrated effectiveness against *E. coli*, *S. aureus*, *Streptococcus* sp., and *Salmonella* sp. The antimicrobial activity of caulerpin was assessed, revealing a MIC of 5.25 mg/mL for *E. coli*, *S. aureus*, and *Salmonella* sp. However, the MIC value against *Streptococcus* sp. was higher, at 15.50 mg/mL [62].

Alteramides are a general term for a class of compounds, and compound **7** shown in Figure 2 is alteramide A [63]. Additionally, marine bacteria, particularly those from the genera *Streptomyces* produce alkaloids like streptomycin (**8**), which exhibit extensive antibacterial and antifungal activities [64]. For example, four indole alkaloids, designated streptoindoles A–D, were obtained from *Streptomyces* sp. ZZ1118, which was cultured on a rice solid medium derived from a gut sample of marine shrimp (*Penaeus* sp.). Streptoindole C showed strong inhibition against *E. coli* and *C. albicans*, with a MIC of 0.007 mg/mL. Streptoindole D displayed weak activity solely against MRSA with an MIC of 0.025 mg/mL. Streptoindoles A and B were effective against all three pathogens, with MIC values ranging from 0.007 to 0.025 mg/mL [65]. These compounds often disrupt essential cellular processes in pathogens, such as protein synthesis, and DNA replication. 

Marine-derived fungi also contribute significantly to the arsenal of bioactive alkaloids with antimicrobial properties. Species from marine environments, such as *Penicillium* and *Aspergillus*, produce various alkaloids which have shown strong antifungal activities against *C. albicans* and *A. niger* [66,67]. The ecological and biochemical diversity of marine organisms presents a valuable resource for developing novel antibacterial and antifungal agents, addressing the growing challenge of antimicrobial resistance. Their unique properties and bioactivities, derived from the challenging marine environments, make them promising candidates for addressing antibiotic resistance and other infectious diseases. 

## 3. Marine-Derived Amino Acids

Marine-derived amino acids represent a rapidly growing area of research in the quest for new antimicrobial agents [68]. These amino acids are produced by a variety of marine organisms, including bacteria, fungi, and algae, and have shown significant antibacterial and antifungal activities [51]. Recent studies have demonstrated the effectiveness of these compounds in combating resistant microbial strains. Halicylindramides are a general term for a class of compounds, and compound **9** shown in Figure 2 is halicylindramide A [69]. Halicylindramides isolated from the Japanese marine sponge *H. cylindruta* exhibited in vitro antifungal activity against *M. ramanniana* at a concentration of 0.0075 mg/disk. Notably, the macrocyclic structure of compounds is crucial for their antifungal properties [70]. Rhodopeptins are a general term for a class of compounds, and compound **10** shown in Figure 2 is Rhodopeptins C1 [71]. Recently, Rhodopeptins C1, C2 C3, C4, and B5 were isolated from *Rhodococcus* sp. These antifungal cyclic lipotetrapeptides consist of a *β*-amino acid and three standard α-amino acids. The Rhodopeptin exhibited in vitro antifungal activity against *C. albicans* with MIC between 0.00125 and 0.005 mg/mL, and against *C. neoformans* with an MIC values ranging from 0.00063 to 0.00125 mg/mL [72]. 

In addition to their antifungal properties, marine-derived amino acids have demonstrated significant antibacterial activities. Four cyclic heptapeptides, identified as L-156,373 and its derivatives, were obtained from a marine *Streptomyces* sp. culture. These heptapeptides showed notable activity against pathogens including *S. aureus*, MRSA, *B. Calmette-Guérin*, and *B. subtilis*, with MIC values ranging from 0.00025 to 0.00125 mg/mL [73]. The continued exploration of marine-derived amino acids and their conjugates could lead to the identification of novel antimicrobial agents with specific activities against resistant bacterial and fungal pathogens. Furthermore, advancements in cultivation and chemical profiling techniques, as demonstrated by the integrated strategy used in these studies, are crucial for the effective discovery and optimization of these bioactive compounds.

## 4. Marine-Derived Peptides

Marine-derived peptides are emerging as potent candidates in combating bacterial and fungal infections, especially given the increasing challenge of AMR [74]. Ilamycins are a general term for a class of compounds, and the name of compound **11** is ilamycin B1 [75]. Cyclic oligopeptides such as ilamycins, sourced from marine bacteria like *Streptomyces atratus*, have shown considerable effectiveness against drug-resistant pathogens including *M. tuberculosis* [76]. Notable examples include kahalalide F (**12**), which is effective against *C. albicans*, and surfactin, which targets MRSA [77]. 

Cyclic oligopeptides are a class of peptides consisting of 2–20 amino acids arranged in a cyclic structure. These peptides are primarily synthesized by non-ribosomal peptide synthetases, which contribute to their structural diversity and biological activity. One notable example is the ilamycin family, which includes ilamycin B1–F and ilamycins G–R. These peptides are produced by the marine-derived bacterium *S. atratus* and have shown potent activity against *M. tuberculosis*. The antimicrobial activity of ilamycins is ascribed to their ability to disrupt bacterial cell walls, making them effective against drug-resistant strains of *M. tuberculosis* [78]. Recent research by Wang et al. [24] has highlighted the structural and functional diversity of cyclic oligopeptides derived from marine microorganisms. The study discusses how modifications in the side chains of amino acids can significantly influence the antimicrobial potency of these peptides. For instance, the presence of specific side chain modifications in ilamycins have been shown to enhance their activity against *M. tuberculosis*, indicating the potential for structural optimization to improve therapeutic efficacy [79]. 

Cyclic depsipeptides are another important class of marine-derived peptides known for their potent antimicrobial properties [80,81]. These peptides contain ester bonds in addition to amide bonds, which contribute to their unique structural characteristics. Compound **13** shown in Figure 2 is didemnin B [82]. Compounds **12** and **13** has demonstrated significant antifungal activity against *C. albicans* [83,84]. The mechanism of action involves disrupting the fungal cell membrane, leading to cell lysis and death.

Cyclic lipopeptides are characterized by the presence of a lipid tail attached to a cyclic peptide core. These compounds exhibit strong surfactant properties, which enhance their ability to disrupt microbial cell membranes [24]. Marine-derived cyclic lipopeptides such as surfactin (**14**) produced by *Bacillus* sp., have shown remarkable antibacterial and antifungal activities. Surfactin has been effective against a range of Gram-positive bacteria, including MRSA. The antimicrobial action of surfactin involves the insertion of the lipid tail into the bacterial cell membrane, leading to increased membrane permeability and cell death [85]. Friulimicin B (**15**) is a natural cyclic lipopeptide composed of eleven amino acids, synthesized by the *Actinoplanes friuliensis*. It exhibits antibacterial activity against a broad range of Gram-positive bacteria, including antibiotic-resistant pathogens [86]. Asperversiamides are cyclic heptapeptides and the name of compound **16** is Asperversiamide A. Asperversiamide A, B and C extracted from a coral-derived fungal strain *Aspergillus versicolor* CHNSCLM-0063, exhibiting strong anti-*M. marinum* activity, with asperversiamide B also showing moderate anti-TB activity [87,88]. Asperheptatides A–B are structurally closest to asperversiamide B and also shows moderate anti-TB activity [89]. By using asperversiamide A as a core structure, various cinnamic acid groups were introduced onto the hydroxyl group of the serine side chain, resulting in cinnamic acid derivatives that exhibited an eight-fold increase in anti-TB action. This process also confirmed the anti-TB efficacy of the cinnamic acid structure [88].

The cyclic peptide marthiapeptide A (**17**) isolated from the deep-sea-derived *Marinactinospora thermotolerans* SCSIO 00652 showed antibacterial MICs of 0.004, 0.002, 0.002, and 0.008 mg/mL for *B. subtilis*, *M. luteus*, *B. thuringiensis*, and *S. aureus*, respectively, and showed no activity against Gram-negative bacteria like *E. coli* [90,91]. Marine-derived peptides often exhibit synergistic effects when combined with conventional antibiotics and antifungals. This synergy can enhance the overall antimicrobial efficacy and reduce the likelihood of resistance development. Some peptides inhibit bacterial efflux pumps, which are responsible for expelling antibiotics from bacterial cells. By blocking these pumps, peptides can restore the potency of existing antibiotics and expand their spectrum of activity. Additionally, marine peptides may target multiple bacterial pathways simultaneously, reducing the chances of resistance [92].

Understanding the structure-activity relationships (SARs) of marine-derived peptides is crucial for optimizing their antimicrobial properties. Structural modifications, such as altering amino acid residues or adding functional groups, can significantly impact the activity and stability of these peptides [93]. For instance, modifications to the side chains of amino acids in ilamycins have been shown to enhance their activity against *M. tuberculosis*. Similarly, altering the lipid tail length in cyclic lipopeptides can improve their ability to penetrate microbial membranes. A wide-ranging review by Wang et al. [24] discusses the SAR of various marine-derived peptides, emphasizing the importance of specific structural features in determining their antimicrobial efficacy. Their review highlights that fine-tuning the peptide structure can lead to the development of more potent antimicrobial properties.

## 5. Marine-Derived Polyketide

Marine-derived polyketides represent a promising frontier in the search for new antimicrobial agents [92]. These compounds, isolated from marine organisms such as bacteria, fungi, and algae, have shown significant potential due to their distinctive characteristics and various ways of combating pathogens. Unlike many terrestrial antibiotics, marine-derived polyketides often possess distinctive chemical features that reduce the likelihood of cross-resistance with existing drugs. Marine-derived polyketides including macrocyclic lactones, polyenes, and polyethers contribute to their potent biological activities and their ability to target pathogens in novel ways [92]. The antimicrobial mechanisms of marine-derived polyketides are varied and can include the inhibition of cell wall synthesis, disruption of membrane integrity, interference with protein and nucleic acid synthesis, and inhibition of critical metabolic pathways. This diversity in modes of action not only enhances their effectiveness but also reduces the risk of resistance development.

Dicitrinones are a general term for a class of compounds, and the compound **18** in Figure 2 is dicitrinone E [94]. Recently, dicitrinones were extracted from the starfish-associated *Penicillium* sp. GGF 16-1-2. These compounds demonstrated potent antifungal properties against *Colletotrichum gloeosporioides*, with LD_50_ values between 0.00958 mg/mL and 0.01614 mg/mL [95]. Five new polyketides (two chromones, two phenyl derivatives, and one tandyukusin derivative) were isolated alongside five known ones. Among these, few showed significant antifungal activities against *Penicillium italicum*. The study underscores the potential of mangrove-derived fungi in yielding bioactive compounds with significant antimicrobial properties [96]. A study by Song et al. [97] focused on aromatic polyketides from the endozoic fungus *Talaromyces minioluteus*, associated with deep-sea cold-seep mussels. Researchers identified five new aromatic polyketides, including a unique benzofuran derivative and four chromone analogs. Compound **19** shown in Figure 2 is talarominine A [97]. Talarominine A and its derivatives demonstrated notable antibacterial activity, particularly against MRSA and *P. aeruginosa*. The study emphasized the distinctive structures of these polyketides, which are essential for developing new antibacterial agents aimed at combating MRSA. Van Anh et al. [98] explored rifamycin-related polyketides from the marine-derived bacterium *Salinispora arenicola*, identifying eight polyketides, including three new derivatives. The rifamycin-related compounds exhibited moderate cytotoxic activity against various cancer cell lines, suggesting potential antibacterial applications due to their ability to inhibit bacterial RNA synthesis.

Aspulvinones are a general term for a class of compounds, and the compound **20** should be aspulvinones H [99]. Recently, four antimicrobial compounds named aspulvinones B’, H, R, and S were isolated from *A. flavus* KUFA1152, sourced from the marine sponge *Mycale* sp. These compounds demonstrate significant antibacterial properties against MREF ATCC 29212 and MRSA *S. aureus* ATCC 29213. In addition to their antibacterial effects, these compounds also prevent biofilm formation by these strains. The MIC values for aspulvinones B’, H, R, and S range between 0.004 and 0.064 mg/mL [100]. A study by Koch et al. [101] isolated 2-carboxymethyl-3-hexylmaleic acid anhydride from the endozoic fungus *Aspergillus tubingensis* OY907, found in the Mediterranean marine sponge *Ircinia variabilis*. This compound demonstrated inhibitory activity against *Neurospora crassa*. Tan et al. [102] emphasized the importance of marine-derived polyketides by identifying novel compounds from the marine-derived fungus *Penicillium* species, showing potent antifungal and antibacterial activities. These compounds were particularly effective against *C. albicans* and *S. aureus*, demonstrating broad-spectrum antimicrobial potential. The study suggested these polyketides could be developed into therapeutic agents for treating bacterial and fungal infections. In addition to their antibacterial properties, marine-derived polyketides show promise in disrupting biofilms, which protect bacterial communities from antibiotics, making infections difficult to treat. Wibowo et al. [26] highlighted the potential of marine-derived bacterial secondary metabolites including polyketides in antibiotic and antibiofilm applications. Their review underscores the importance of marine bacteria as a valuable resource for discovering new antimicrobial agents.

## 6. Marine-Derived Naphthoquinones

Marine-derived naphthoquinones are characterized by their naphthalene ring structure with two ketone groups. Isolated from various marine organisms, including sponges, algae, and bacteria, these compounds exhibit a wide range of biological activities [103]. Marine-derived naphthoquinones demonstrate a wide range of antimicrobial activities. They often disrupt the electron transport chain in microbial cells, hindering ATP production and causing cell death. Some naphthoquinones also intercalate into DNA, blocking replication and transcription, which prevents microorganisms from reproducing and functioning properly. Additionally, these compounds can induce the formation of ROS within microbial cells, leading to oxidative damage to proteins, lipids, and DNA, and resulting in cellular dysfunction and death [51,104,105]. 

Mersaquinone (**21**), a newly discovered derivative of tetracene, was isolated from *Streptomyces* sp. EG1, obtained from sediment off Egypt’s North Mediterranean coast. The chemical structure of mersaquinone was meticulously identified using HRESIMS, IR spectroscopy, and both one-dimensional and two-dimensional NMR spectroscopy. This substance demonstrated the ability to suppress the growth of the MRSA strain TCH1516, showing a MIC of 0.00336 mg/mL [106]. Marine sponges are rich sources of naphthoquinones. Compounds such as avarone (**22**) and avarol (**23**), isolated from *Dysidea avara*, have demonstrated potent antibacterial and antifungal activities. These compounds have shown effectiveness against marine bacteria (*C. marina*, *M. stanieri*, *V. fischeri*, and *P. haloplanktis*) and marine fungi (*H. mediosetigera*, *A. cruciatus*, *L. uniseptate*, and *M. pelagica*) [107]. Naphthazarins are a general term for a class of compounds, and compound **24** shown in Figure 2 is 5,8-Dihydroxy-1,4-naphthoquinone [108]. These compounds are mostly effective against a range of bacterial and fungal pathogens. Recent studies have demonstrated the significant antibacterial potential of marine-derived naphthoquinones, such as those isolated from *Streptomyces* and *Talaromyces* sp. For example, Park et al. [109] identified two new naphterpin derivatives from *Streptomyces* sp. CNQ-509, which exhibited potent antibacterial activity against several pathogens. The study highlighted the role of the naphthoquinone moiety in the antibacterial efficacy of these compounds. Flaviogeranins are a general term for a class of compounds, and compound **25** shown in Figure 2 is flaviogeranin B. Shen et al. [110] reported the isolation of a group of naphthoquinone-containing compounds, specifically seven flaviogeranin (**25**) congeners, including three novel compounds, from *Streptomyces* sp. B9173. Their structures were determined by a combination of spectroscopic techniques, including 1D and 2D NMR, and high-resolution mass spectrometry. The compounds flaviogeranin B and flaviogeranin C2 exhibited potent inhibitory activity against *S. aureus* and *M. smegmatis*, with MIC values ranging from 0.005 to 0.009 mg/mL, comparable to the MIC of the positive control, erythromycin.

Phorbaketals are a general term for a class of compounds, and compound **26** shown in Figure 2 is phorbaketals A. Kim et al. [111] explored the antibiofilm properties of phorbaketals (**26**) extracted from the Korean marine sponge *Phorbas* sp. against *S. aureus*, including MRSA. The study isolated six different phorbaketals (phorbaketal A, A acetate, B, B acetate, C, and C acetate) and tested for their ability to prevent biofilm formation. Specifically, compounds B and C demonstrated notable antibiofilm activity without bactericidal effects, thereby minimizing the potential for resistance. These compounds also suppressed the production of staphyloxanthin, a virulence factor aiding *S. aureus* in evading the immune system. The antibiofilm action was attributed to the downregulation of crucial genes associated with biofilm formation and virulence, including α-hemolysin (*hla*) and nuclease (*nuc1*). Consequently, phorbaketals B and C show promise as potential antibiofilm agents for treating infections caused by drug-resistant bacteria. Another recent study by Lee et al. [112] demonstrated the antibiofilm activity of collismycin C (**27**), a compound isolated from the marine bacterium *Streptomyces* sp. MC025, against MRSA. The study involved screening 79 Micronesian marine microorganisms for their ability to inhibit *S. aureus* biofilm formation. Among the isolated compounds, collismycin C was the most effective, inhibiting MRSA biofilm development by over 90% at 0.05 mg/mL. The biofilm suppression activity of collismycin C was attributed to its iron-chelating properties, which interfere with the biofilm formation process. In addition to their antibacterial properties, marine-derived naphthoquinones also exhibit strong antifungal activities, addressing the critical need for new antifungal agents amidst rising drug resistance. Liu et al. [113] studied naphthoquinone derivatives from the mangrove-derived endophytic fungus *Talaromyces* sp., identifying twelve 1,4-naphthoquinone derivatives, including two new compounds. These derivatives showed significant antifungal effects against *C. albicans* and *A. niger*, with some compounds demonstrating higher potency than standard antifungal agents. The study emphasized the dual functionality of these naphthoquinones in addressing both bacterial and fungal infections.

## 7. Marine-Derived Terpenoids

Marine-derived terpenoids are characterized by their diverse and complex structures, often consisting of multiple isoprene units. Marine-derived terpenoids frequently display unique modifications such as halogenation, glycosylation, and unusual ring systems. These modifications arise from the distinctive biosynthetic pathways found in marine environments, contributing to the enhanced biological activities of these compounds [114]. Marine-derived terpenoid derivatives are increasingly recognized for their potent antibacterial and antifungal activities, making them a promising frontier in the development of new antimicrobial agents. Their unique bioactivities and mechanisms of action make them valuable candidates for novel therapies to combat resistant microbial strains and biofilm-associated infections. Marine-derived terpenoids can disrupt microbial cell membranes, prevent cell wall synthesis, restrict protein and nucleic acid synthesis, and inhibit critical metabolic pathways. Additionally, some terpenoids can modulate the immune response, enhancing the host’s ability to fight off infections. For instance, compounds like squalene and its derivatives have been shown to disrupt microbial membranes, leading to cell lysis and death.

Terpenoid derivatives isolated from marine sponges have demonstrated strong antimicrobial properties. Compound **28** shown in Figure 2 is strobilactone A [115]. Four drimane-type sesquiterpenoid lactones were extracted from the fungus *A. insuetus* (OY-207), which was sourced from the Mediterranean sponge *Psammocinia* sp. These compounds were identified as derivatives of strobilactone A, including a new compound identified as (E)-6-(4′-hydroxy-2′-butenoyl)-strobilactone A. In addition, the known derivatives strobilactone A and (E,E)-6-(6′,7′-dihydroxy-2′,4′-octadienoyl)-strobilactone A, along with 2α,9α,11-trihydroxy-6-oxodrim-7-ene, were also isolated. The antifungal properties of these compounds were tested, revealing that strobilactone A and (E,E)-6-(6′,7′-dihydroxy-2′,4′-octadienoyl)-strobilactone A exhibited mild antifungal activity against the fungus *Neurospora crassa* [116].

Terpenoids from marine fungi and algae have revealed considerable effectiveness against fungal pathogens like *C. albicans* and *A. niger*. Research on the endophytic fungus *P. chrysogenum* QEN-24s, isolated from the marine red alga *Laurenica* sp., resulted in the discovery of two new polyoxygenated sterols, penicisteroid A and B, along with the previously known steroid, anicequol. These compounds were evaluated for their antifungal properties. The findings revealed that penicisteroid A (**29**) exhibited antifungal activity against both *A. niger* and *Alternaria brassicae*, while anicequol was active only against *A. brassicae*. In contrast, penicisteroid B showed no antifungal activity against either of the two fungi [117]. Generally, terpenoid derivatives often disrupt fungal cell membranes or inhibit ergosterol synthesis, a critical component of fungal cell membranes, leading to fungal cell death. This dual functionality underscores the versatility and broad-spectrum potential of marine-derived terpenoids in antimicrobial therapy.

Marine-derived terpenoid derivatives have shown promise in addressing biofilm-associated infections. Terpenoid derivatives have demonstrated the ability to disrupt biofilm formation and maintenance. By targeting the biofilm matrix and preventing the development of new biofilms, these compounds offer a viable solution for treating biofilm-related infections. This characteristic is particularly valuable in medical settings, where biofilm formation on medical devices and implants poses significant challenges. Three novel sesterterpenes belonging to the ophiobolin class were isolated from the marine-derived fungus *Emericella variecolor* strain GF-10, which was sourced from sediment collected at a depth of 70 meters in the Gokasyo Gulf, Japan. These compounds were identified as ophiobolin K, 6-epi-ophiobolin K, and 6-epi-ophiobolin G. They were found to inhibit the biofilm formation of *M. smegmatis*, with ophiobolin K (**30**) demonstrating the highest activity, showing a MIC of 1.84 mg/mL [118].

## 8. Marine-Derived Polysaccharides 

**Figure 3:**
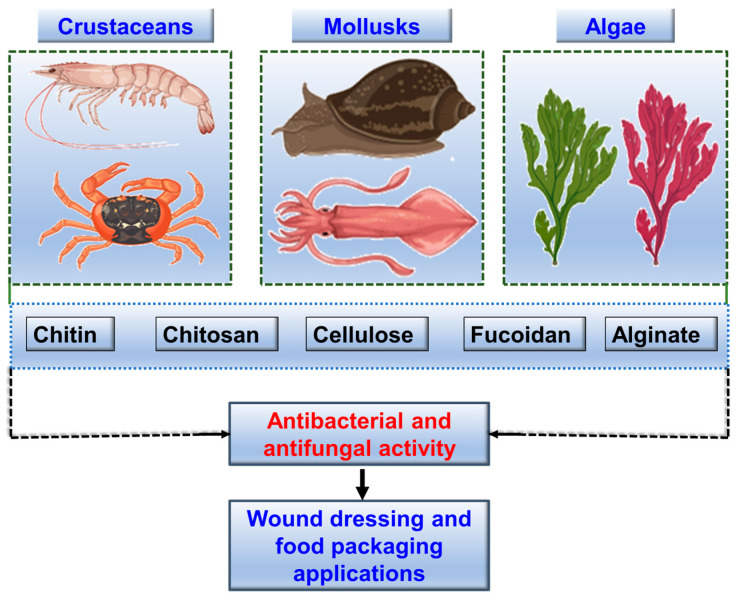
The sources of selected key biopolymers and their applications. Crustaceans and mollusks provide chitin, chitosan and cellulose. Algae (such as green and red algae) provide cellulose, fucoidan and alginate. These biopolymers exhibit antibacterial and antifungal activities, making them suitable for use in wound dressing and food packaging applications.

Marine-derived polysaccharides are complex carbohydrates that consist of long chains of monosaccharide units. These polysaccharides often display unique structural features such as sulfation, branching, and acetylation, which contribute to their diverse biological activities. Marine-derived polysaccharides have garnered significant interest for their antibacterial and antifungal properties, leading to their exploration in various industrial applications, from pharmaceuticals to food packaging. Marine-derived polysaccharides can inhibit microbial adhesion and biofilm formation, disrupt cell membranes, and modulate immune responses. Additionally, some marine polysaccharides have been shown to enhance the efficacy of existing antibiotics, potentially reducing the required dosage and minimizing side effects. Recent studies have highlighted the effectiveness of key marine polysaccharides such as chitin, chitosan, cellulose, fucoidan, and alginate. Figure 3 schematically illustrates an overview of the key marine polysaccharides and their sources from marine organisms.

Chitin is a polymer consisting of (1→4)-β-linked N-acetyl-D-glucosamine units. It is the most abundant amino-polysaccharide polymer found in nature and ranks as the second most abundant polysaccharide after cellulose. Marine ecosystems, particularly crustacean shells from crabs, shrimp, lobsters, and krill, are considered the primary sources of chitin [119]. A study by Abdel-Rahman isolated highly pure chitin from Brazilian Atlantic Coast shrimp shells by the chemical treatment method. The isolated chitin exhibited antibacterial activity against *E. coli* by the chemo-luminescence technique. Chitosan, derived from the deacetylation of chitin found in the exoskeletons of crustaceans, has been extensively studied for its antimicrobial properties [120,121]. Its polycationic nature allows chitosan to interact with negatively charged bacterial membranes, causing cell leakage and death. Recent research has demonstrated the effectiveness of chitosan in food packaging and biomedical applications. A recent study by Mohammadi et al. [122] extracted chitosan from shrimp waste using different methods: conventional extraction and microwave-assisted extraction. The antibacterial action of the extracted chitosan was evaluated using an agar disc diffusion assay. The chitosan obtained through conventional extraction displayed the highest antibacterial zone of inhibition (mm) against *Listeria monocytogenes* (9.48), *E. coli* (8.79), and *S. Typhimurium* (8.57). On the other hand, chitosan produced via microwave-assisted extraction showed superior activity against *S. aureus* (8.05) and *E. coli* (8.37) with a similar antibacterial property against *L. monocytogenes* (6.52) and *S. Typhimurium* (7.34). A recent study by Verma et al. [123] demonstrated a process of extracting chitosan from the shells of horse mussels, a common waste product from fisheries. The extracted chitosan displayed a degree of acetylation of 57.43%, making it a suitable biopolymer for biomedical applications. The research highlights the antimicrobial efficacy of the chitosan, particularly against *E. coli* and *B. subtilis*, demonstrating its potential as a valuable material in antimicrobial applications.

Marine-derived cellulose and nanocellulose from algae such as *Ulva lactuca* shows significant antibacterial properties [124]. This cellulose is a promising antibacterial agent against *K. pneumonia* (ST627), *S. aureus* (ATCC6538), *E. coli* (ATCC25922) and coagulase-negative *staphylococci*. It disrupts bacterial membranes and adsorbs onto cells, effectively inhibiting growth. Its biocompatibility and antibacterial nature also make it suitable for wound dressings and food packaging, promoting sterility and extending shelf life [125,126,127]. Ongoing research aims to enhance these properties, making marine-derived cellulose a sustainable antibacterial material. Fucoidan is a type of sulfated polysaccharide found mainly in various species of brown seaweed and some marine invertebrates [128,129]. Fucoidan is primarily composed of fucose, a type of sugar, along with sulfate groups. It may also contain other monosaccharides like glucose, galactose, and mannose. Recent studies have shown its effectiveness against various bacterial pathogens, including *S. aureus*, *E. coli*, *L. monocytogenes*, and *Salmonella enterica serovar Typhimurium*. The antibacterial activity of fucoidan is attributed to its ability to disrupt bacterial cell walls and membranes, inhibit bacterial adhesion and biofilm formation, and interfere with bacterial metabolism. Its sulfate groups interfere with the replication of viruses and the growth of bacteria. Additionally, fucoidan has shown potential against fungal pathogens such as *C. albicans* by impairing cell wall integrity and preventing adhesion and biofilm formation [127,129]. Carrageenan, extracted from algae, is known for its antibacterial and antifungal properties [130]. It forms protective barriers on surfaces, inhibiting the adherence and penetration of pathogens. This polysaccharide is widely used in the food industry as a natural preservative to enhance product shelf life by preventing microbial growth [130].

Alginate is a naturally occurring biopolymer derived primarily from the cell walls of brown seaweed and contain linear copolymers of α-L-guluronic acid and β-D-mannuronic acid. Alginate forms gels in the presence of calcium ions and exhibits notable antimicrobial properties [131]. Alginate-based wound dressings are highly absorbent and maintain a moist atmosphere favorable to healing while protecting against microbial infections. In the food industry, alginate is used as a thickener and stabilizer, with its antimicrobial properties enhancing food safety and quality [132]. Recent developments in composite films combining marine-derived polysaccharides have shown enhanced antimicrobial efficacy. For instance, *O*-carboxymethyl and pectin films, when combined with neem extracts, demonstrated significant antimicrobial activity and improved tensile properties [133]. Such composite films are biodegradable and offer a sustainable solution for food packaging and other applications requiring antimicrobial properties. 

Overall, a variety of marine-derived substances have demonstrated potential as antibacterial and antifungal agents. Table 1 provides a comprehensive overview of various marine-derived compounds, including their type, sources, properties, and modes of action.

## 9. Conclusions

In conclusion, the rising threat of antimicrobial resistance (AMR) necessitates the discovery of novel antimicrobial agents. Our review highlights the untapped potential of marine environments, which harbor a diverse array of bioactive compounds. Marine-derived alkaloids, peptides, polyketides, naphthoquinones, terpenoids, and polysaccharides exhibit significant antibacterial and antifungal properties against resistant pathogens. Notable compounds such as zamamidine D, manzamine A, ilamycins, and kahalalide F show particular promise. The unique mechanisms of action and potential for synergistic effects with existing antibiotics make natural marine products a valuable resource in the fight against AMR. Future research should focus on the exploration and sustainable utilization of these marine-derived compounds to develop new, effective antimicrobial therapies.

## 10. Future Prospectives

The future of marine-derived antimicrobial research lies in several key areas:

(a) Advanced Bioprospecting and Sustainable Harvesting

Advancements in bioprospecting technologies, including accessing previously unexplored marine habitats such as deep-sea vents and polar regions, can lead to the discovery of novel bioactive compounds. Emphasizing sustainable harvesting methods, including microbial fermentation and aquaculture, will minimize environmental impact and ensure a continuous supply of these valuable compounds.

(b) Synthetic Biology and Genome Mining

Synthetic biology offers the potential to enhance the production and structural diversity of marine-derived compounds. Genome mining techniques can identify new biosynthetic pathways and gene clusters responsible for producing bioactive metabolites, leading to the discovery of new compounds and the optimization of existing ones for improved antimicrobial efficacy.

(c) Structural Optimization and Drug Development

Understanding the SAR of marine-derived compounds is crucial for optimizing their antimicrobial properties. Structural modifications, such as altering amino acid residues or functional groups, can enhance the potency, stability, and bioavailability of these compounds. Collaborative efforts between chemists, biologists, and pharmacologists are essential to translate these compounds into clinically viable drugs.

(d) Combination Therapies

Exploring the synergistic effects of marine-derived compounds with conventional antibiotics and antifungals can enhance overall antimicrobial efficacy and reduce resistance development due to different targets of drugs. Combination therapies could be particularly effective against multidrug-resistant pathogens and biofilm-associated infections.

(e) Ecological and Conservation Considerations

Preserving marine biodiversity is paramount for maintaining the source of these bioactive compounds. Conservation efforts should focus on protecting marine ecosystems and mitigating the impacts of climate change, pollution, and overfishing. Additionally, understanding the ecological roles of these compounds can provide insights into their evolutionary significance and potential applications.

(f) Clinical Trials and Regulatory Approval

Rigorous preclinical and clinical trials are necessary to evaluate the safety, efficacy, and pharmacokinetics of marine-derived compounds. Establishing regulatory frameworks will facilitate the approval and commercialization of new marine-based antimicrobial agents.

(g) Toxicity Issues

Marine-derived compounds hold therapeutic potential but pose toxicity risks such as cytotoxicity, neurotoxicity, hepatotoxicity, and immunotoxicity. Future strategies to mitigate these issues include advanced bioprospecting to identify safer compounds, synthetic biology for producing less toxic analogs, and structural optimization to improve selectivity. Rigorous preclinical and clinical testing, combination therapies to reduce doses, and comprehensive regulatory frameworks are essential.

## Figures and Tables

**Table 1:** List of marine-based drugs, outlining their characteristics and methods of action.

Type	Compounds	Marine-Sources	Properties	Mode of Action
Marine-derived alkaloids	Zamamidine D	*Amphimedon* sp.	Antibacterial and antifungal activity [42]	Inhibition of topoisomerase IV and bacterial DNA gyrase and membrane disruption, inhibition of ergosterol synthesis, and disruption of fungal cell wall.
	Manzamine	*Acanthostrongylophora* sp.	Antibacterial activity [134]	Inhibition of bacterial cell wall synthesis.
	Bromoageliferin	*Agelas dilatata*	Antibacterial activity [49]	Inhibition of bacterial protein synthesis, disruption of membrane integrity and preventing biofilm formation.
	Caulerpin	*Caulerpa* sp.	Antibacterial activity [62]	Disruption of bacterial cell membranes, and inhibition of enzymatic activity.
	Streptoindoles	*Streptomyces* sp.	Antibacterial activity [65]	Inhibition of protein synthesis and interruption of membrane integrity, DNA binding and interference.
Marine-derived amino acids	Halicylindramides	*Halichondria cylindruta*	Antifungal activity [70]	Alteration in membrane integrity, reserve enzymatic activity, and induction of oxidative stress.
	Rhodopeptins	*Rhodococcus* sp.	Antifungal activity [72]	Disruption of cell membrane integrity, inhibition of enzymatic activity, and induction of oxidative stress.
Marine-derived peptides	Ilamycins	*Streptomyces atratus*	Antibacterial activity [76]	Disruption of protein synthesis and inhibition of RNA synthesis.
	Kahalalide F	*Elysia rufescens*	Antifungal activity [83]	Destabilization of cell membrane, induction of apoptosis, and inhibition of enzymatic activity.
	Didemnins	*Trididemnum* sp	Antibacterial activity [55]	Inhibition of DNA synthesis, interference with protein synthesis, and induction of apoptosis in bacteria.
	Friulimicin	*Actinoplanes friuliensis*	Antibacterial activity [86]	Inhibtion of cell wall synthesis and interaction with cell membranes.
	Asperversiamides	*Aspergillus versicolor*	Antibacterial activity [87,88]	Damage to membrane integrity, suppression of protein synthesis, and inhibition of cell wall synthesis.
	Marthiapeptide	*Marinactinospora thermotolerans*	Antibacterial activity [91]	Damage to membrane integrity, inhibition of protein synthesis, and inhibition of cell wall synthesis.
Marine-derived polyketides	Dicitrinones	*Penicillium* sp.	Antifungal activity [95]	Disruption of cell membrane integrity, inhibition of enzymatic activity, and induction of oxidative stress.
	Talarominine	*Talaromyces minioluteus*	Antibacterial activity [97]	Inhibition of protein synthesis, damage to cell membrane integrity, and inhibition of nucleic acid synthesis.
	Aspulvinones	*Aspergillus flavus*	Antibacterial activity [100]	Disruption of cell membrane integrity, inhibition of enzymatic activity, and induction of oxidative stress.
Marine-derived naphthoquinones	Mersaquinone	*Streptomyces* sp.	Antibacterial activity [106]	Inhibition of protein synthesis, disruption of cell membrane integrity, and inhibition of DNA synthesis.
	Avarone	*Dysidea avara*	Antibacterial and antifungal activity [107]	Disruption of cell membrane integrity, prevention of enzymatic activity, and induction of oxidative stress.
	Naphterpin	*Streptomyces* sp.	Antibacterial activity [109]	Disruption of cell membrane integrity, and disruption of cell wall synthesis.
Marine-derived terpenoids	Penicisteroid	*Penicillium chrysogenum*	Antifungal activity [117]	Disruption of cell membrane integrity, inhibition of enzymatic activity, and induction of oxidative stress.
	Ophiobolin K	*Emericella variecolor*	Antibacterial activity [104]	Disruption of cell membrane, inhibition of proteins, enzymes and DNA, and generation of ROSs.
Marine-derived polysaccharides	Chitin	Shrimp shell	Antibacterial activity [121]	Compromise of membrane integrity, and disruption of cell wall synthesis.
	Chitosan	Crustaceans	Antibacterial and antifungal activity [119,121]	Cell membrane disruption, suppression of protein and DNA synthesis, and chelation of essential nutrients
	Cellulose	*Ulva lactuca*	Antibacterial activity [124]	Compromise of cell membrane integrity, generation of ROSs and inhibition of enzymatic activity.
	Fucoidan	Brown algae	Antibacterial and antifungal activity [128,129]	Disruption of cell membrane integrity, inhibition of enzymatic activity, and binding to bacterial surface structures.
	Alginate	Seaweed	Antibacterial activity [131,132]	Disruption of cell membrane integrity, inhibition of enzymatic activity, and induction of oxidative stress.

## Data Availability

Data are contained within the article.
